# CoFUSE-EPWNet: Cross-Modal Consistency Fusion for RGB-D Express Packaging Waste Detection in Green Campus Management

**DOI:** 10.3390/s26144396

**Published:** 2026-07-10

**Authors:** Fei Yu, Gufeng Gong, Liujun Li

**Affiliations:** 1 School of Education, Teesside University (In Partnership with Amity Global Institute), Singapore 238853, Singapore; e4445979@live.tees.ac.uk; 2 School of Computer Science and Mathematics, Central South University of Forestry and Technology, Changsha 410004, China; t20090523@csuft.edu.cn; 3 Department of Soil and Water Systems, University of Idaho, Moscow, ID 83844, USA

**Keywords:** multimodal, computer vision, express packaging waste, waste detection

## Abstract

The rapid growth of express packaging waste (EPW) is creating increasing challenges for urban waste management and high-quality resource recovery, while reliable automated sorting remains difficult because of heterogeneous materials, deformable packaging, random stacking, and frequent partial occlusion. This study develops a system-oriented RGB-D sensing framework to improve the reliability of EPW sorting in conveyor-belt recycling operations. A synchronized and spatially registered RGB-D paired dataset, MEPWaste, was constructed containing 4528 paired samples (9056 RGB/depth images) from 10 representative categories of inner and outer packaging under multiple levels of stacking and occlusion. Based on this dataset, a multimodal detection framework was designed to improve feature reliability within each modality, reinforce spatial consistency between modalities, and enhance detection stability in densely stacked scenes with fragmented visible evidence. The proposed framework achieved 89.1% ${mAP}_{50}$ and 69.3% ${mAP}_{50:95}$, exceeding the RGB-only model by 5.3 and 5.8 percentage points, respectively, and the RGB-D baseline by 2.7 and 3.4 percentage points, with recall reaching 85.1%. A closed-loop sensing–inference–execution pipeline integrating synchronized RGB-D acquisition, real-time detection, robotic grasping, and PLC-based control was further implemented to examine engineering feasibility. Overall, the study provides a practical technical pathway for improving EPW sorting reliability and supporting intelligent resource recovery in waste management systems, with potential applicability to green campus management scenarios characterized by high-frequency parcel consumption and concentrated packaging waste generation.

## 1. Introduction

With the intensification of environmental pollution and the escalation of climate-related risks, improving the sustainability, operational efficiency, and recovery quality of MSW governance systems has emerged as a primary focus in global environmental governance and circular economy strategies [1]. In this context, the persistent growth of e-commerce and the swift expansion of parcel delivery networks have significantly heightened both the intensity of generation and the growth rate of EPW, which is increasingly recognized as a rapidly growing contributor to urban MSW streams, characterized by rapid expansion, spatial dispersion, and notable material diversity [2]. In China, the amount of express deliveries increased from 95.77 billion parcels in 2022 to 155.57 billion in 2024, representing an approximate growth of 62.4% [3]. The swift expansion has led to increasing pressures on urban sanitation systems and downstream recycling value chains, as the demands for packaging reduction, resource recovery, and environmentally responsible disposal extend from logistics growth into municipal operations and the wider secondary materials sector [4]. Campus environments provide a representative application scenario for EPW governance because parcel collection points, dormitory areas, and teaching facilities generate concentrated packaging waste with diverse materials and irregular disposal patterns. Therefore, reliable automated EPW detection can serve as a technical foundation for green campus management by enabling more accurate sorting, recyclable-material recovery, and data-driven waste management.

The governance issues related to EPW surpass its swiftly increasing production volume and are primarily driven by its multifaceted complexity concerning material characteristics, structural configurations, and operational circumstances [5,6]. Common packaging materials, such as corrugated cardboard boxes, plastic bags, bubble wrap, document envelopes, and expanded polystyrene containers, demonstrate significant variability in mechanical strength, surface reflectance, structural integrity, and contamination levels [7,8]. In real recycling environments, collecting terminals and sorting facilities typically exhibit high-throughput turnover, mixed stacking, random occlusion, and time-constrained handling, resulting in highly unstructured operational conditions [9]. Taken together, these factors make source separation more difficult and create uncertainty in obtaining recyclable materials with sufficient purity. Although EPW contains valuable resources, including paper fibers, recyclable polymers, and foam-based materials [10], these resources can only be effectively recovered when different packaging types are identified and separated with adequate precision. So, the accuracy of sorting not only affects the recognition performance, but also the stability of the downstream recycling process. Automated identification and sorting systems, which are designed to be reliable and feasible in terms of engineering, can help improve the quality of EPW recycling and facilitate a more efficient recovery system [11,12].

However, the existing EPW sorting and recovery methods are still mainly labor-intensive and semi-mechanized, and are subject to the problems of low sorting efficiency, high labor intensity, sorting quality and rising labor costs [13]. Consequently, their capacity to handle an increased amount of EPW to be processed and recovered is decreasing. Many studies have explored the application of object detection based on deep learning to solid-waste recognition and automated sorting guidance to enhance the sorting efficiency. End-to-end feature learning, as in the detectors YOLO [14] and Faster R-CNN achieves more effective visual representations and generally outperforms the traditional detectors based on hand-crafted features or rule-based matching [15]. EPW scenes, however, are especially difficult due to the diversity of materials, occluded objects, and reflective and semi-transparent surfaces that can interfere with RGB imaging [16]. With random stacking and partial occlusion, RGB appearance alone can be insufficient to describe the boundaries of objects, separate the appearance of similar categories or infer the spatial relationships between objects that overlap in the image. These issues can result in missed detection, false alarms and class confusion, while reducing the robustness of RGB-only-based detection in real sorting stations.

In recent years, multimodal methods for detecting have been investigated to compensate for some of the drawbacks of RGB-only vision, and include using geometric information like depth data in addition to RGB images [17]. In comparison to color appearance alone, depth information can convey extra structural information in the presence of unclear textures and/or unstable illumination, which can be beneficial in stacked and occluded scenes for separating and locating objects. But the use of multimodal detection for EPW sorting is still in a relatively nascent stage and is plagued with some practical difficulties [18]. Because RGB and depth data are acquired at different resolutions and from different viewpoints, and have varied noise patterns and underlying statistical distributions, it is hard to cross-modally align data. When the two are concatenated or linearly fused with fixed weights, the two modalities may fail to make good use of each other’s complementary information and might even inject depth noises or spatial errors into the features to be fused. This is quite a severe issue in practice in real sorting stations where irregular stacking, mixed materials and partial occlusion can lead to depth holes and biased geometric responses. These inconsistencies, along with synchronization errors and viewpoint changes between the sensors, can lead to higher false detection rates and missed detection rates. Furthermore, practical deployment is limited by the constraints on computing power, power consumption and real-time processing, and the deployment of multimodal models must be accompanied by consideration of the inference efficiency. Another drawback is that many of the current approaches primarily aim at feature fusion, and have not been concerned about the processing of the fragmented visible parts in the prediction head. This can result in box drift, object adhesion and unstable classification under heavy occlusion.

Although RGB-D detection and multimodal feature fusion have been widely explored in object detection and waste recognition, existing methods are usually designed for relatively stable object categories or general multimodal perception scenarios. In express packaging waste recycling and sorting, however, the detection problem has several task-specific difficulties. Packaging materials are often stacked, folded, compressed, semi-transparent, or partially occluded. In these cases, RGB appearance cues may suffer from weak texture, reflection, and boundary ambiguity, while depth maps may contain missing regions, noisy responses, or unstable structural information. Therefore, directly concatenating RGB and depth features or applying generic multimodal fusion strategies may not sufficiently address the modality inconsistency caused by cluttered and occluded packaging waste scenes.

To address this gap, this study proposes CoFUSE-EPWNet, a cross-modal consistency fusion framework for RGB-D express packaging waste detection. The novelty of the proposed method lies not simply in the use of RGB-D input, but in its task-oriented fusion mechanism for unreliable and inconsistent multimodal cues. Specifically, DAPG introduces depth-aware prior guidance to enhance structural reliability, CPDF performs progressive cross-modal dynamic fusion to improve RGB–depth consistency under occlusion, and MS-SEAMHead strengthens multi-scale spatial enhancement and attention-guided localization for deformable and boundary-ambiguous packaging materials. The main contributions of this study are summarized as follows:

(1) A multimodal express packaging waste dataset, MEPWaste, is constructed for campus recycling and sorting scenarios. The dataset contains paired RGB and depth images, ten packaging categories, multi-label co-occurrence, stacking, and different levels of occlusion.

(2) A cross-modal consistency fusion network, CoFUSE-EPWNet, is proposed for RGB-D express packaging waste detection. Different from simple early fusion, late fusion, or direct feature concatenation, the proposed framework explicitly models the reliability and consistency of RGB and depth features under cluttered and occluded scenes.

(3) Three task-oriented modules, namely DAPG, CPDF, and MS-SEAMHead, are designed to improve depth-aware structural guidance, progressive RGB–depth feature interaction, and multi-scale spatial localization, respectively. These modules are particularly suitable for detecting deformable, reflective, and boundary-ambiguous packaging materials.

(4) Extensive experiments are conducted to evaluate detection accuracy, inference efficiency, category-level performance, occlusion robustness, and prototype sorting feasibility. The results demonstrate that CoFUSE-EPWNet improves detection performance while maintaining practical potential for edge-side sorting.

## 2. Related Work

### 2.1. Object Detection Algorithms for Waste and Express Packaging Waste Detection

For the last few years, many object detection algorithms based on deep learning have been studied and applied to recognize, locate, and sort solid waste [19]. Currently, there are two-stage and one-stage detectors. Two-stage detectors such as R-CNN [20] and Faster R-CNN [21] can be relied upon to perform well in terms of localization in complex scenes. But there is extra computational expense for their steps of proposal generation, classification, and regression, making them unsuitable for use in a high throughput sorting line where real-time processing is needed. In contrast, one-stage detectors like SSD [22] and YOLO [23] series perform classification and regression of the bounding-box in a single forward pass. This enhances their inference speed without compromising accuracy, making them more practical for real-world waste sorting tasks.

Recent research has mainly enhanced the YOLO-based detectors with attention modules, feature fusion strategies and optimized detection heads for solid-waste scenes with occlusion, stacking, cluttered background, and dense small targets. Meanwhile, increasingly more investigations have focused on the feasibility of these models in real sorting workflows, instead of just reporting performance on single test images. Demetriou, et al. [15] developed and published a construction and demolition waste (CDW) dataset, systematically comparing one-stage and two-stage detectors for real-time localization and classification. Their findings indicate that YOLOv7 provides an advantageous speed–accuracy balance, while Faster R-CNN demonstrates greater robustness in complex stacking and adhesion scenarios. Recently, Ma, et al. [24] created an image-near-infrared spectral paired dataset for throwaway package waste (FDPWaste) and introduced a collaborative detection and material-recognition framework to validate multimodal identification in sorting applications. In cases with significant occlusion, such as electronic garbage, Liu, et al. [25] developed the OEWaste mixed-image dataset, which includes systematic occlusion-level annotations, and improved target separation and recognition under occlusion by incorporating DyHead and CPCA into the detection framework. Collectively, these studies suggest that most gains are achieved via stronger feature representation, improved multi-scale aggregation, and head-level refinement; however, the majority of methods still rely primarily on RGB appearance cues, and occlusion is often treated implicitly rather than being modeled as a first-class source of failure.

Despite these progressions, the majority of current works continue to heavily exploit RGB appearance cues and the scene category they focus on is either a waste category with relatively stable material properties or a relatively structured scene layout. EPW sorting, on the contrary, is characterized by the presence of material heterogeneities, the prevalence and presence of films that are transparent/semitransparent and reflective polymers, high structural variability, and frequent random stacking and occlusion. With these conditions, RGB-only detectors are easily failed in many aspects: e.g., wrong object boundaries, wrong categorization of similar visual appearances, and wrong estimations of spatial relationships between overlapped objects. The presence of other objects or cluttered backgrounds in the scene can cause interference—although it is possible to reduce some of this interference through the use of attention modules or more refined detection heads—in the absence of geometric information and if the uncertainty caused by occlusion is not taken into account when preparing the data or designing the model. It is therefore important that EPW detection should not only be seen as a sub-task of waste detection. It calls for perception and modeling strategies that are able to reliably integrate complementary information from multiple sources, while aligning them.

### 2.2. Multimodal Detection and Fusion for Waste Identification

Due to the limitations of RGB image sensing, recent investigations into solid-waste detection have included other sensing modalities, such as depth, non-invisible spectrum (e.g., near infrared), and hyperspectral imaging, to represent the properties of materials, the geometry of objects, and occlusion relationships in complex sorting scenarios [26,27]. These modalities offer information that cannot be easily derived from color images alone, and may aid in enhancing the object separation and stability of object detection for automated sorting.

In the depth-assisted detection point of view, RGB–depth fusion can help alleviate ambiguous cues in structure when estimating boundaries and understanding spatial relationships, particularly when objects are stacked or occluded. Most of the current literature works with RGB and depth features at the feature level, for instance, the two types of features are concatenated, added element-wise, or interacted with each other across modalities. The actual advantage of fusion, however, crucially relies on the quality of the depth map, the correctness of the image registration and the distribution gap between the two modalities. Simple fusion methods may also result in false responses and a decrease in detection stability, if the depth maps are noisy or incomplete, or if they are not synchronized correctly [16,17,28].

This is particularly important for plastic waste, as some materials are difficult to distinguish in RGB images due to similar colors, reflective surfaces, or transparency. Liu, et al. [29] proposed a dual-source multi-scale fusion method based on MSI–RGB for fine-grained plastic waste sorting, where band selection and feature compression were used to reduce spectral redundancy and improve material-level discrimination. Ji, et al. [30] built an RGB–HSI multimodal instance segmentation dataset for plastic waste identification and highlighted the value of hyperspectral information for distinguishing materials with similar visual appearances. Cai, et al. [31] further developed a dual-sensor acquisition system combining RGB and hyperspectral cameras, together with an asymmetric multi-scale fusion network. In their method, spectral–spatial features are extracted from the hyperspectral branch after dimensionality reduction, and then fused with the RGB feature pyramid to improve detection performance across different models. BANet has recently demonstrated the effectiveness of bidirectional feature aggregation and adaptive multi-scene perception for robust lane detection in autonomous driving. Although BANet focuses on lane perception rather than RGB-D waste detection, it provides useful evidence that adaptive feature aggregation can improve perception robustness in complex scenes. In contrast, CoFUSE-EPWNet further addresses the modality inconsistency between RGB and depth information in stacked and occluded express packaging waste scenarios.

While multimodal perception has been proven to be beneficial for several solid-waste detection tasks, it is not an easy task to introduce it to real EPW sorting stations. First, many existing methods still require coarse weighting or direct concatenation of the RGB and depth features, that is, RGB and depth features are assumed to be well aligned in space and semantics. In real EPW scenes, however, the wrinkled packaging, the mixtures of materials, irregular stacking often lead to the occurrence of depth holes, noisy responses and spatial bias, and these errors may be amplified in the process of fusion. Second, the reliability of each modality under occlusion is not explicitly taken into account and unreliable channels or local regions can affect the fused representation. Third, a majority of methods rely on the traditional prediction head and address the visible evidence sparsely due to occlusion only superficially. This may cause box drift, object stickiness and unstable classification. Occlusion and mixed materials are more serious issues in high throughput EPW sorting where the model is required to be able to do real-time inference whilst sorting. Thus, to better address the multimodal scenario, intra-modal reliability as well as spatial consistency prior to fusion and local visible evidence at the prediction stage should be taken into account.

Overall, previous studies have demonstrated the effectiveness of RGB-D perception, multimodal feature fusion, and deep learning-based waste detection. However, most existing fusion strategies mainly focus on improving general feature representation through early fusion, late fusion, attention-based fusion, or transformer-based interaction. These methods do not fully consider the specific failure modes in express packaging waste sorting, where RGB and depth modalities may become unreliable in different ways due to stacking, deformation, reflection, transparency, and severe occlusion.

In particular, soft cushioning materials such as air bags, bubble film, EPE foam, and gas column bags often exhibit weak texture, unstable shape, and ambiguous boundaries. At the same time, depth images may suffer from missing or noisy responses when these materials are compressed, overlapped, or partially occluded. Therefore, a simple fusion strategy may introduce redundant or misleading information rather than improving detection robustness. This motivates the development of a task-oriented cross-modal consistency fusion framework that can selectively enhance reliable modality cues, progressively align RGB–depth representations, and improve spatial localization under cluttered packaging waste scenes.

Compared with existing RGB-D or multimodal waste detection methods, CoFUSE-EPWNet focuses on the consistency and reliability of multimodal cues rather than only increasing the number of input modalities. The proposed DAPG, CPDF, and MS-SEAMHead modules are designed to address depth-aware structural guidance, progressive cross-modal interaction, and multi-scale spatial prediction, respectively, thereby improving detection robustness for stacked, occluded, and deformable express packaging waste.

## 3. Method

### 3.1. Research Design

In this paper, according to the main difficulties in practical EPW sorting, the research design comprises dataset construction, model development and evaluation, and system-level validation. This design explores multimodal perception for EPW sorting from three angles: whether the data correspond to the real sorting process, whether the model has a positive effect on detection, and whether this method can be applied in an engineering application.

(1) Dataset construction

An industrial RGB camera and a depth camera are installed above a conveyor belt to collect paired RGB and depth images under realistic conveying and stacking conditions. The two sensors are synchronized so that the captured images maintain temporal consistency and spatial correspondence. Based on this acquisition setup, the MEPWaste dataset is constructed. It contains 4528 aligned RGB–depth pairs, corresponding to 9056 images, and covers 10 representative categories of inner and outer express packaging. Instance-level bounding-box annotations are provided to support reproducible benchmarking of multimodal detection methods.

(2) Methodology and result analysis

CoFUSE-EPWNet is developed as a cooperative RGB–depth fusion network for improving detection robustness in stacked and occluded EPW scenes. The model is evaluated under unified experimental settings by comparing it with representative two-stage detectors and lightweight one-stage detectors. In addition, ablation experiments are conducted to analyze the contributions of RGB-only, depth-only, and RGB–depth inputs, as well as the effects of key modules, including intra-modal feature enhancement, spatial selection, and patch-aware prediction refinement.

(3) Intelligent detection and sorting system

The intelligent sorting system consists of three main modules: multimodal acquisition, real-time detection, and sorting execution. In this system, synchronized sensing and online inference produce detection results, which are then converted into sorting actions to form a closed-loop perception-to-decision process. Validation in a realistic sorting environment shows that the proposed multimodal detection approach can support intelligent EPW recovery in practical conveyor-belt applications.

### 3.2. Dataset Construction

**Figure 1:**
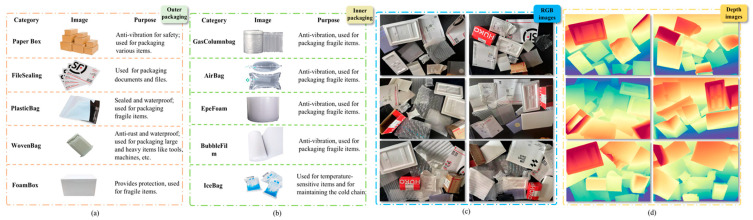
The MEPWaste datasets: (**a**) express outer packaging; (**b**) express inner packaging; (**c**) RGB image samples; (**d**) depth image samples.

The category design and annotation rules are based on the following several standards: the Beijing Specification for Green Express Packaging Utilization and Assessment [32], the Technical Specification for Pollution Control in the Classification and Recycling of Express Packaging Waste [33], and the Classification and Coding of Express Packaging [34]. These standards can serve as a guideline to define packaging types and to address multi-label situations in complex situations. The RGB-D sensing system (i.e., RGB camera and depth camera) is used for data collection. The two cameras take simultaneous RGB and depth images to ensure temporal consistency and spatial correspondence between RGB and depth images. Multimodal feature fusion and robust detection is thus based on this setup, as a data basis, in unstructured sorting environments (as shown in Figure 1c,d).

(1) Data source and acquisition-scene construction

To make the dataset better reflect campus express packaging waste recycling and sorting scenarios, this study simulated typical scenes such as campus courier stations, centralized pickup areas near dormitories, and temporary recycling points around teaching buildings. The data collection was conducted by three researchers over a period of four weeks. Before image acquisition, the researchers collected and organized common express packaging waste samples in campus environments according to the predefined category system. These samples included outer packaging materials such as paper boxes, file-sealing bags, plastic bags, woven bags, and foam boxes, as well as inner cushioning materials such as gas column bags, air bags, EPE foam, bubble film, and ice bags. All samples were basically cleaned and checked before acquisition to avoid obvious damage, severe contamination, or ambiguous categories that could affect subsequent annotation quality.

The MEPWaste dataset was collected in controlled indoor prototype scenes that were constructed to simulate typical campus express packaging waste recycling and sorting conditions. It should be noted that the dataset was not collected from a continuously operating industrial sorting line. Instead, the acquisition scenes were designed by referring to common campus courier stations, centralized pickup areas near dormitories, temporary recycling points around teaching buildings, and preliminary sorting platforms. This setting allowed the researchers to control category composition, stacking patterns, occlusion levels, and RGB-D acquisition quality while preserving the main visual characteristics of campus express packaging waste sorting scenarios.

During acquisition-scene construction, different packaging categories were randomly arranged on the collection platform with varying positions, orientations, stacking heights, and category combinations. These arrangements were used to simulate mixed states such as centralized disposal after parcel pickup, temporary stacking in dormitory areas, and cluttered scenes before preliminary sorting at recycling points. Therefore, MEPWaste should be understood as a controlled RGB-D prototype dataset for campus express packaging waste detection, rather than a dataset directly collected from long-term real-world sorting operations.

**Figure 2:**
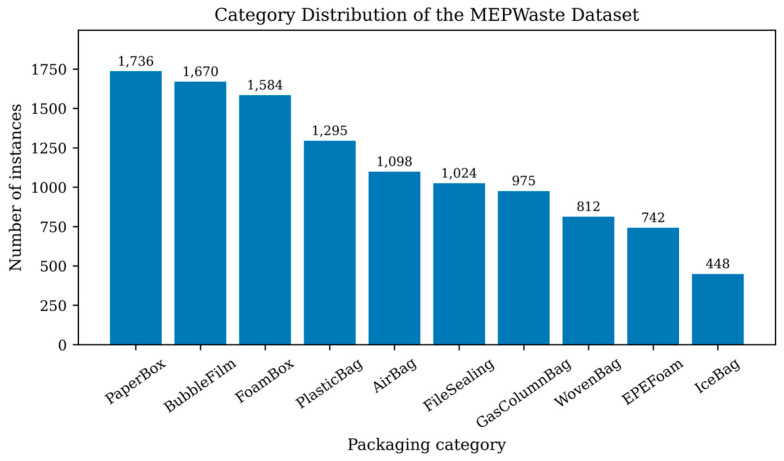
Category distribution of the MEPWaste dataset.

MEPWaste contains 4528 paired RGB–depth samples, with 9056 images in total, including 4528 RGB images and 4528 depth images. The dataset covers various object shapes and complex stacking scenes commonly found in campus express packaging waste recycling and sorting scenarios. According to the industry classification standards for inner and outer express packaging, the samples were divided into 10 categories. The outer packaging categories include PaperBox, FileSealing, PlasticBag, WovenBag, and FoamBox; the inner packaging types include GasColumnBag, AirBag, EPEFoam, BubbleFilm, and IceBag, as shown in Figure 1a,b. The category distribution is imbalanced, which reflects the actual occurrence frequency of different packaging materials in campus express packaging waste and also indicates their practical value in recycling-oriented sorting. Among them, FoamBox, BubbleFilm, and PaperBox have the largest number of instances, with more than 1500 instances each, while IceBag has a relatively smaller number of instances, approximately 450. The category distribution of MEPWaste is shown in Table 1 and Figure 2. The dataset contains 11,384 annotated object instances in total. Among the ten categories, PaperBox, BubbleFilm, and FoamBox contain relatively large numbers of instances, with 1736, 1670, and 1584 instances, respectively. In contrast, IceBag has the smallest number of instances, with 448 annotated objects. This imbalanced distribution reflects the actual occurrence frequency of different express packaging materials in campus recycling and sorting scenarios. To reduce the potential influence of category imbalance on model evaluation, category information was further considered during stratified dataset partitioning, and per-category detection performance is reported in the experimental section.

(2) RGB-D image acquisition and data processing

Data acquisition was carried out using an RGB-D sensing system composed of a Basler acA1920-40gc industrial RGB camera and an Intel RealSense D435i depth camera, which were used to acquire RGB images and depth images, respectively. The Basler industrial RGB camera was mainly used to capture color, texture, edge, and appearance information of packaging waste, while the Intel RealSense D435i depth camera was used to obtain spatial structure, relative height, and stacking relationship information of the targets. The acquisition devices were fixed above or obliquely above the collection area to simulate the installation perspective of campus intelligent recycling equipment, conveyor-belt sorting systems, or fixed visual detection devices. During acquisition, the distance between the cameras and the collection platform was kept stable and adjusted appropriately according to the stacking height and field of view, ensuring that the target area could be fully covered. Image acquisition was conducted under controlled indoor lighting conditions while referring to common illumination environments in campus courier stations and recycling points, so as to reduce the influence of strong illumination changes and preserve realistic visual characteristics of campus recycling scenes. Although the controlled indoor lighting condition helped reduce severe imaging noise and ensured the quality of RGB-D registration, it also indicates that the dataset may not fully cover all visual disturbances in open or long-term recycling environments. In real campus recycling points or industrial sorting stations, stronger illumination variation, dust, stains, background clutter, object motion, and continuous material flow may introduce additional challenges. Therefore, the current dataset is mainly intended to evaluate RGB-D multimodal detection under controlled but cluttered prototype sorting conditions.

For each acquisition, the two cameras were triggered synchronously to capture RGB and depth images at the same time, forming one paired RGB–depth sample and ensuring temporal consistency between the two modalities. After acquisition, the researchers conducted an initial screening of the raw images and removed samples with image blur, severe target loss, abnormal depth information, or obvious mismatch between RGB and depth images. Then, camera calibration and image registration were performed on the retained samples. Specifically, the intrinsic and extrinsic parameters of the industrial RGB camera and depth camera were first obtained through calibration. Based on these calibration parameters, the depth images were spatially mapped to align with the RGB images at the pixel level as much as possible. After calibration and registration, each RGB image corresponded to one spatially matched depth image, providing a reliable data basis for subsequent multimodal feature fusion and occlusion-robust detection.

To cover typical stacking and occlusion patterns, samples were collected from different viewpoints and heights, and samples with different degrees of occlusion were intentionally included. Three occlusion levels were defined in the dataset: light, moderate, and heavy occlusion. Occlusion coverage was considered during both acquisition and sample screening. During dataset curation, the occlusion level was assigned according to the degree to which the target instance was covered by other objects, namely the degree of visible information loss under stacking conditions. Light occlusion indicates that most of the target area is visible, with only local edges or corners occluded; moderate occlusion indicates that the main body of the target remains recognizable, but some key appearance information is covered by other objects; heavy occlusion indicates that a large part of the target is occluded, leaving only limited visible regions, which requires recognition based on local texture, edge morphology, or depth structure. In this way, MEPWaste reflects common recognition challenges in campus express packaging waste recycling and sorting, including multi-label co-occurrence, object overlap, and severe occlusion.

To improve the reproducibility of occlusion-oriented evaluation, the number of samples and object instances under each occlusion level was further counted. As shown in Table 2, MEPWaste contains 1628 RGB-D pairs with level-1 occlusion, 1837 RGB-D pairs with level-2 occlusion, and 1063 RGB-D pairs with level-3 occlusion. In terms of annotated object instances, level-3 occlusion includes 3194 instances, accounting for 28.06% of all annotated objects. In the test set, 638 object instances belong to level-3 occlusion, accounting for 28.03% of the test instances. These statistics indicate that the test set retains a sufficient number of heavily occluded samples and can support reproducible evaluation of occlusion robustness (see Table 3).

MEPWaste provides bounding-box annotations and category labels for all samples in cluttered backgrounds, supporting the training and evaluation of multi-object detection and multi-label recognition models. Some images naturally contain multi-label co-occurrence, significant overlap, and obvious occlusion, thereby forming a more challenging detection benchmark. By preserving detailed visual information from RGB images together with spatial structural information from depth images, MEPWaste provides a challenging and engineering-relevant benchmark for campus recycling scenarios, supporting multimodal feature fusion, occlusion-robust detection in unstructured environments, and intelligent sorting of express packaging waste.

(3) Dataset partitioning and stratified sampling strategy

To improve the reproducibility of model training and evaluation, the MEPWaste dataset was divided into training, validation, and test subsets at the RGB-D pair level. Each RGB image and its corresponding depth image were treated as an inseparable sample unit during dataset partitioning. Therefore, the two modalities of the same sample were always assigned to the same subset, avoiding cross-modal data leakage.

The 4528 RGB-D pairs were divided into training, validation, and test sets at a ratio of 7:1:2. Specifically, 3170 RGB-D pairs were used for model training, 453 RGB-D pairs were used for validation and hyperparameter tuning, and 905 RGB-D pairs were used for final testing. The test set was not involved in model selection or parameter adjustment.

To further reduce the risk of data leakage caused by highly similar scenes, samples collected from the same acquisition sequence, the same stacking layout, or visually similar consecutive frames were not split across different subsets. In addition, a stratified sampling strategy was adopted to maintain comparable distributions of packaging categories and occlusion levels among the training, validation, and test sets. For each RGB-D pair, the dominant packaging category and the maximum occlusion level among annotated instances were used as stratification indicators. This strategy ensures that each subset contains representative samples from different packaging categories and different occlusion conditions, especially heavily occluded scenes (see Table 4).

### 3.3. The CoFUSE-EPWNet Model

EPW is not always neatly arranged, clean, free from tearing, and free from contamination or obstruction in real recycling and sorting situations. These conditions render the information to be drawn from the visual and/or geometric data incomplete and not always reliable. Consequently, one-modality detectors can have difficulty providing consistent recognition in sorting scenes with high levels of occlusion. Occlusion, stains, and surface damage are a few factors that can make RGB images subject to uncertainty, potentially compromising the integrity of texture information. However, depth images contain valuable geometric information, but are not as well suited when describing material properties and fine boundary details.

**Figure 3:**
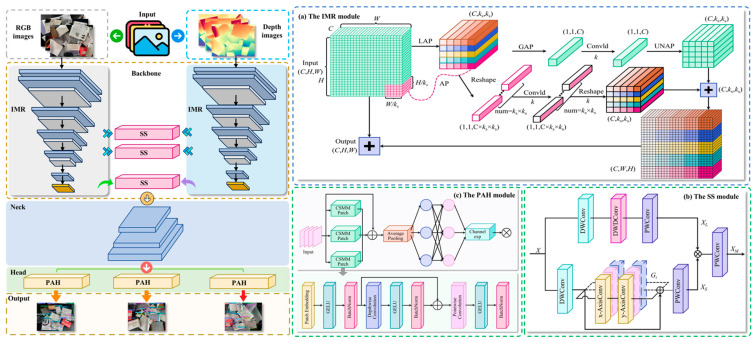
Schematic illustration of improvements provided by the CoFUSE-EPWNet model: (**a**) IMR module; (**b**) SS module; (**c**) PAH module.

Even when RGB and depth data are used together, stacking and occlusion can still cause spatial inconsistency between the two modalities and produce redundant or noisy feature responses. If these features are fused directly, the errors may be carried into multi-scale feature construction and further affect prediction stability (see Figure 3). At the detection head, fragmented visible regions and local mixing between adjacent objects can also interfere with classification and bounding-box regression. Therefore, EPW sorting under severe occlusion requires a multimodal detection framework that improves feature reliability, maintains spatial consistency, and strengthens prediction stability at the same time.

This paper introduces CoFUSE-EPWNet, a cross-modal collaborative fusion detection system, motivated by these factors. As illustrated in Figure 3, the framework implements an end-to-end, three-stage collaboration process, guided by the principles of intra-modal reliable feature encoding, spatial consistency selection, and prediction-stage modeling of partial visibility (IMR–SS–PAH). Initially, RGB and depth images are processed by two structurally aligned, parameter-independent backbone branches, which ensure cross-modal scale correspondence and facilitate modality-specific hierarchical feature extraction. The IMR is employed during encoding to enhance dependable channel responses in the presence of occlusion and to diminish unreliable features (Figure 3a). Secondly, before the neck network, the SS is utilized to explicitly represent spatial correspondences between modalities, selecting, filtering, and aligning prominent regions across various scales. This stage alleviates spatial ambiguity and fusion noise caused by stacking and occlusion, thereby yielding more consistent fused representations for multi-scale feature construction (Figure 3b). Finally, the PAH module is integrated into the multi-scale detection head. By combining multi-scale patch representations with lightweight channel-spatial joint modulation, fragmented visible areas are converted into more stable discriminative evidence, reducing local misalignment and feature intermixing caused by occlusion (Figure 3c).

#### 3.3.1. Dual-Branch Multimodal Backbone with Intra-Modal Hierarchical Recalibration

In practical recycling and sorting contexts of EPW, targets often display irregular stacking, partial damage, and significant occlusion, resulting in insufficient visual information and rendering both textural and structural cues very vulnerable to disruption [35,36]. In such intricate circumstances, a singular modality finds it challenging to deliver consistent and distinctive representations. The RGB modality is especially susceptible to occlusion, contamination, and fluctuations in light, whereas the depth modality, albeit being comparatively resilient in geometric representation, has a constrained ability to depict material qualities and intricate details [37]. This study develops a dual-branch multimodal feature-encoding backbone to leverage the complementing capabilities of various modalities under occlusion and incorporates a hierarchical channel self-recalibration mechanism within the backbone, resulting in a cohesive multimodal feature encoding module.

As illustrated in Figure 3a, the proposed multimodal backbone network consists of two structurally aligned yet parameter-independent feature extraction branches, designed to process RGB and depth image inputs, respectively. The two branches are strictly aligned in terms of network architecture and hierarchical configuration, each being constructed through a progressive stacking of convolutional units and hierarchical feature extraction modules. This design enables the synchronous generation of multi-level semantic feature representations across different scales. Let the input of modality m∈{r,d}  be denoted as $I^{m}$; the feature representation extracted by the backbone at the l-th hierarchical level is given by:

(1) $$F_{l}^{m}=\varepsilon_{l}^{m}\left(F_{l-1}^{m}\right),\;{\;F}_{l}^{m}\epsilon R^{C_{l}\times H_{l}\times W_{l}},\;\;l=1,\ldots ,L$$

where $\varepsilon_{l}^{m}(\cdot )$ denotes the encoding mapping of modality m at the l-th hierarchical level, and the two branches are aligned in the spatial scale, i.e., $H_{l}^{r}=H_{l}^{d}$ and $W_{l}^{r}=W_{l}^{d}$. This alignment ensures spatially consistent feature representations across modalities at each semantic level, thereby providing a stable foundation for subsequent cross-modal interaction.

Considering that the discriminative contributions of different channels vary substantially across feature hierarchies under occlusion, a hierarchical channel self-recalibration mechanism is introduced at the backbone encoding stage to dynamically modulate channel responses through a collaborative scheme that balances global semantic consistency and local visibility sensitivity. For the feature representation at the l-th hierarchical level $F_{l}^{m}$, a global channel descriptor is first constructed as:

(2) $$z_{l}^{m,g}=GAP(F_{l}^{m})$$

In parallel, a locally aware channel descriptor is constructed as:

(3) $$z_{l}^{m,l}={LAP}_{K_{l}}(F_{l}^{m})$$

where GAP(⋅) denotes global average pooling, and ${LAP}_{K_{l}}(\cdot )$ denotes local average pooling after partitioning the feature map into $K_{l}$ local regions, which is used to explicitly aggregate locally observable structural information under occlusion. Subsequently, the global and local descriptors are jointly modeled via a hierarchy-dependent fusion coefficient $\lambda_{l}\;$ to generate the channel modulation weights as:

(4) $$\alpha_{l}^{m}=\sigma (G_{l}\left(\lambda_{l}z_{l}^{m,g}+(1-\lambda_{l})z_{l}^{m,l}\right))$$

where $G_{l}(\cdot )$ denotes a lightweight channel mapping function at the l-th hierarchical level, whose output dimension is consistent with the channel number $C_{l}$, and σ(⋅) denotes the Sigmoid activation function, ensuring that $\alpha_{l}^{m}\in [0,1{]}^{C_{l}}$ serves as a channel-wise modulation weight vector.

(5) $${\tilde{F}}_{l}^{m}=\alpha_{l}^{m}⨀F_{l}^{m}$$

It is worth emphasizing that the above channel self-recalibration process is performed independently within each modality branch (intra-modal). Specifically, $\alpha_{l}^{m}$ is estimated separately for the RGB and depth features and applied to recalibrate them individually, thereby enhancing the feature reliability of each modality under occlusion without introducing any cross-modal interaction.

The suggested channel self-recalibration mechanism is intrinsically integrated into the feature encoding process of the dual-branch backbone, facilitating the concurrent execution of feature selection and feature extraction. At shallow feature levels, there is a pronounced focus on augmenting locally discernible details to alleviate detail loss due to occlusion, while at deeper feature levels, the mechanism emphasizes global semantic coherence and background suppression to enhance the resilience of object structure representation amidst stacking and cluttered backgrounds. The integration of the dual-modality backbone and the hierarchical channel self-recalibration mechanism enables the multimodal feature encoding module to generate more stable and distinctive feature representations in complex occlusion scenarios, thus supplying high-quality inputs for subsequent cross-modal gated fusion and patch-aware prediction.

#### 3.3.2. The Multi-Scale Guided Spatial Selection Module

After completing intra-modal feature encoding and channel reliability enhancement, although the semantic quality of the representations from different modalities has been substantially improved, in real-world EPW sorting scenarios, the prevalence of random stacking, partial occlusion, and irregular object shapes means that informative cues from different modalities may still exhibit spatial inconsistency and fragmentation. Such spatial misalignment directly undermines the stability of subsequent feature aggregation and multi-scale feature pyramid construction.

Therefore, prior to feeding features into the neck network, it is necessary to introduce an explicit spatial modeling mechanism to guide and filter spatial responses across different scales. By explicitly modeling inter-modal spatial correspondences before the neck, the proposed mechanism selects spatially consistent and complementary responses, thereby reducing occlusion-induced spatial ambiguity and enhancing fusion stability.

This study introduces a multi-scale guided spatial selection mechanism between the corresponding layers of the dual-modality backbone to model and align spatial saliency prior to fusion. Let the features extracted from the corresponding backbone layers of the two modalities be denoted as $X^{r},X^{d}\in R^{C\times H\times W}$; a set of scale-dependent spatial perception operators is first applied to construct multi-scale spatial representations:

(6) $$X^{\left(m,s\right)}=S^{\left(s\right)}\left(X^{m}\right),\;m\in \left\{r,d\right\},\;s=1,\ldots ,S$$

where $S^{\left(s\right)}(\cdot )$ denotes the spatial mapping operator at the s-th scale, which extracts local details, structural contours, and larger-scale contextual layout information under different receptive field ranges. Through multi-scale spatial modeling, the feature representations can better adapt to the spatial variations of express packaging waste under different stacking configurations and degrees of occlusion.

To avoid introducing redundant interference caused by the naive aggregation of multi-scale spatial responses, scale-guided spatial selection weights are further introduced to adaptively filter spatial responses at different scales. Specifically, for each scale-specific feature $X^{\left(m,s\right)}$, a corresponding spatial selection weight is constructed as:

(7) $$W^{\left(m,s\right)}=\sigma (H^{\left(s\right)}\left(X^{\left(m,s\right)}\right))$$

where $H^{\left(s\right)}(\cdot )$ denotes a lightweight spatial mapping function, and σ(⋅) is the Sigmoid activation function. The resulting weight map characterizes the relative importance of spatial locations at the current scale, thereby suppressing background regions and noise responses under occlusion while highlighting spatially discriminative regions.

Guided by the spatial selection weights, the multi-scale spatial features are weighted and fused, and a spatially enhanced representation is generated under inter-modal consistency constraints as follows:

(8) $$\tilde{X}=P\left(\left(\sum_{s=1}^{S}W^{\left(r,s\right)}⊙X^{\left(r,s\right)}\right)\right)∥\left(\left(\sum_{s=1}^{S}W^{\left(d,s\right)}⊙X^{\left(d,s\right)}\right)\right)$$

where ⊙ denotes element-wise multiplication, ∥ denotes feature concatenation, $X^{\left(r,s\right)}$ represents the spatial feature representation of the RGB modality at the s-th scale, and $X^{\left(d,s\right)}$ represents the spatial feature representation of the depth modality at the s-th scale. P(⋅) denotes a point-wise mapping function, which is used to integrate the feature representations guided by spatial selection.

Through the proposed multi-scale guided spatial selection mechanism, the model is able to adaptively focus on spatial regions of express packaging waste that are discriminative under complex occlusion conditions. At smaller scales, the mechanism emphasizes local detail cues such as edges, folds, and damage, while at larger scales it preserves the ability to capture the overall stacking structure and contextual layout. Functionally, this mechanism is complementary to the intra-modal feature encoding stage: the former concentrates on inter-modal spatial consistency selection and alignment, whereas the latter focuses on enhancing intra-modal feature reliability. Together, they improve the stability of cross-modal fusion and multi-scale feature pyramid construction, and provide the subsequent prediction head with more coherent spatial representations.

#### 3.3.3. The Patch-Aware Prediction Head Module

Following the execution of intra-modal reliable feature encoding and inter-modal spatial consistency selection, the prediction step of EPW remains significantly challenged by severe occlusion and dense stacking. The visible areas of things are frequently fractured, and local intermingling between neighboring items is prevalent. These problems may result in feature misalignment, entanglement of local information, and the loss of critical discriminative cues during classification and localization regression within the detection head.

We additionally implement a patch-aware prediction augmentation approach at the detection head to resolve this issue. The suggested module enhances prediction resilience in occlusion-dominated circumstances by integrating multi-scale patch modeling with joint channel-spatial mixing, transforming locally visible patches into more stable discriminative evidence.

Let the feature input to the prediction enhancement module be denoted as $X\in R^{C\times H\times W}$. Considering the scale variations of visible regions caused by occlusion, the module parallely adopts multiple patch partition scales $\left\{p_{1},p_{2},p_{3}\right\}$ to perform region-wise representation of the feature, and extracts responses through channel–spatial mixing units, yielding multi-patch head outputs $\left\{U_{p}\right\}$. The multi-head responses are then aggregated to form a region-enhanced intermediate representation:

(9) $$U=\sum_{p\in \{p_{1},p_{2},p_{3}\}}C_{p}(X)$$

where C denotes the number of channels of the input feature, and H and W denote the spatial height and width of the feature map, respectively; U denotes the region-enhanced intermediate representation obtained by aggregating the multi-patch head outputs; and $C_{p}(\cdot )$ denotes the channel–spatial mixing mapping function associated with patch scale p, which takes X as input and outputs the corresponding patch-aware response. This multi-head aggregation enables simultaneous coverage of fine-grained local textures and larger-scale structural contours, thereby alleviating scale instability under random stacking and occlusion in EPW scenarios.

To capture spatial–channel correlations without significantly increasing computational overhead, $C_{p}(\cdot )$ adopts a lightweight depth-wise separable convolutional residual structure to model local spatial patterns, and combines it with point-wise convolution to enable cross-channel information fusion. Its core formulation can be abstracted as:

(10) $$C_{p}\left(X\right)=X+PW(DW\left(PE_{p}\left(X\right)\right))$$

where ${PE}_{p}(\cdot )$ denotes patch embedding, which maps local regions to embedded representations according to patch scale p; DW(⋅) and PW(⋅) denote depth-wise convolution and point-wise convolution, respectively (optionally followed by lightweight normalization and activation functions). This formulation enhances the structural responses of visible regions through localized convolutions, while the point-wise mapping compensates for inter-channel interactions, avoiding the loss of cross-channel information that typically arises from purely channel-wise convolutions.

After aggregating the multi-patch heads, the module generates channel-wise weights via global aggregation and introduces an exponential mapping to expand the dynamic range of the weights, thereby improving tolerance to occlusion-induced local misalignment and response bias. Specifically, the enhanced feature is computed as:

(11) $$\tilde{X}=exp(\sigma \left(MLP\left(GAP\left(U\right)\right)\right))⊙X$$

where GAP(⋅) denotes global average pooling, MLP(⋅) is a lightweight channel mapping, σ(⋅) is the Sigmoid activation, and exp(⋅) monotonically maps the weight range from $\left[0,1\right]$ to (1,e] to amplify the modulation capacity. The operator ⊙ denotes channel-wise scaling. The resulting $\tilde{X}$ is then fed into the classification and regression branches of the detection head, enhancing consistent modeling of visible fragments and global object structure under occlusion.

Considering the wide scale variation and diverse occlusion levels of EPW objects, the proposed patch-aware prediction enhancement module is embedded into a multi-scale detection head and applied at three detection scales. This design simultaneously addresses small-scale fragmented visibility and large-scale holistic structures. Without significantly increasing computational cost, it strengthens the association between occluded targets and non-occluded regions, reduces background interference on classification and localization, and ultimately improves model robustness in complex stacking and occlusion-dominated sorting scenarios.

## 4. Experiment Environment and Evaluation Metrics

During deep learning training, careful tuning of key hyperparameters is essential to ensure model effectiveness and accuracy when handling complex images [38,39]. Detailed information on the experimental environment and parameter settings is provided in Table 5.

To quantitatively evaluate the performance of the proposed model in express packaging waste detection, this study adopts IoU as the fundamental evaluation metric. IoU measures the degree of overlap between the predicted region and the ground-truth annotation, and is defined as follows:

(12) $$IoU=\frac{Area(predicted\;region\;\cap \;ground\;truth\;region)}{Area(predicted\;region\;\cup \;ground\;truth\;region)}$$

In object detection tasks, IoU quantifies the degree of overlap between the predicted bounding box and the ground-truth bounding box, and serves as the basis for computing mAP [40]. mAP evaluates detection performance by calculating the area under the P-R curve, and is defined as follows:

(13) $$mAP=\frac{1}{C}\sum_{c=1}^{c}AP_{c},AP_{c}=\int_{0}^{1}P_{c}\left(R_{c}\right)dR_{c}$$

where C denotes the total number of express packaging waste categories, and $AP_{c}$ represents the average precision for the c-th category.

This study employs ${mAP}_{50}$ (mAP at an IoU threshold of 0.5, %), ${mAP}_{50:95}$ (the mean mAP averaged over IoU thresholds from 0.5 to 0.95, %), precision (%), and recall (%) to evaluate the accuracy and completeness of express packaging waste detection. The formulations of precision and recall are defined as follows:

(14) $$Precision=\;\frac{TP}{TP+FP},Recall=\;\frac{TP}{TP+FN}$$

where TP denotes the number of true positives, FP denotes the number of false positives, and FN denotes the number of false negatives.

## 5. Experiment Results and Analysis

### 5.1. Experimental Results

**Figure 4:**
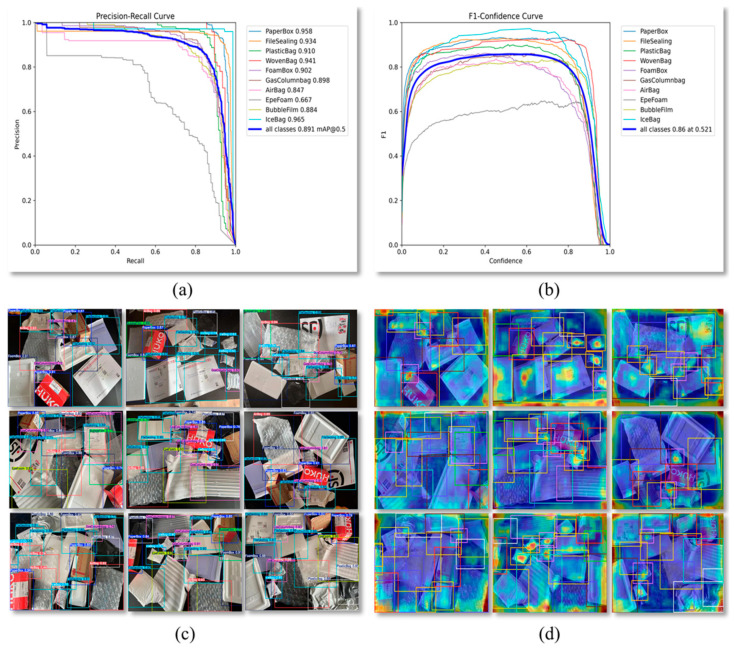
Detections and heatmaps of the CoFUSE-EPWNet model: (**a**) precision–recall curve; (**b**) F1–confidence curve; (**c**) detections of the MEPWaste dataset under different occlusions; (**d**) heatmap of the MEPWaste dataset under different occlusions.

Figure 4a displays the P-R curves of the model for the multi-class occlusion detection task, encompassing both inner and outer EPW packaging categories. The curves exhibit stable precision envelopes across a wide recall range, resulting in an overall performance of ${mAP}_{50}$ = 89.1%, signifying effective practical applicability in sorting scenarios characterized by stacking, occlusion, and background interference. The results also suggest that different categories are affected by occlusion to different degrees.

Categories with stable shapes and clear boundaries, such as rigid outer packaging, keep relatively high precision over a wide range of recall values. This indicates that a strong structure assists the model to boost recall without introducing many false positive. In contrast, soft inner packaging and areas where the boundaries have been poorly defined reveal a distinct approximation of the P-R envelope, as well as a rapid reduction in precision with an increase in recall. These categories can thus be considered as being more occlusion sensitive. This difference is very closely related to material and shape features. There may be a tendency for soft or film packaging to be distorted when stacked and for the edges to become fuzzy or obscured. This causes the visible evidence to become more scattered, spatial ambiguity to be greater, and the false positive and false negative to be harder to maintain. Therefore, these occlusion sensitive categories are used as critical test cases in the following comparative and ablation experiments.

The F1–confidence curve is presented in Figure 4b for various confidence values. This curve is used for analyzing the overall precision and recall. The higher the confidence level, the F1 score first improves and then deteriorates until it achieves the optimal value of 86.0% with a confidence level of 52.1%. This finding suggests that a moderate level of confidence is optimal for the model’s precision–recall trade-off and does not require an extreme threshold. This behavior is helpful for actual sorting systems where static or slightly changed thresholds are desired.

When the confidence threshold becomes higher, the F1 score decreases mainly be-cause occlusion-sensitive categories tend to receive low-to-medium confidence scores. In stacked and occluded scenes, only a portion of the object may be visible, thus the model may have less evidence to make predictions. This indicates that the performance restrictions during occlusion are not only associated to localization/classification errors but also to unstable prediction confidence. This is a finding that, from the modeling point of view, gives the idea that for detection problems where occlusion dominates we need feature enhancement and the prediction-aware design.

To show the detection results under light, moderate and heavy occlusion in real EPW sorting scenes, representative results are shown in Figure 4c. Under light occlusion, the model produces compact detections that are consistent with the object categories, with few false positives or missed targets. This shows that recognition is stable when sufficient visual and geometric evidence is available. When occlusion increases to a moderate level, visible regions become more fragmented, but the detector still preserves object integrity and localization for most targets, especially for outer packaging with clearer structures. This indicates that the model can tolerate partial visibility caused by stacking. Under heavy occlusion, although only small object fragments may remain visible and different items may overlap with each other, the model is still able to detect many targets and provide reasonable localization for visible instances. This suggests that the proposed design can make use of sparse local evidence instead of being dominated by background or surrounding objects.

Figure 4d further shows the feature response heatmaps under different occlusion levels. Under light occlusion, the high-response areas are mainly located on the key structural parts of EPW objects. Under moderate occlusion, the responses remain concentrated on visible edges, folds, and geometric discontinuities, indicating that the model can still use local discriminative cues when complete object information is unavailable. Under severe occlusion, response distributions become more dispersed and may partially overlap with neighboring objects; however, the model continues to emphasize structurally informative regions rather than being dominated by background noise, suggesting a degree of object-consistent representation even under extreme occlusion.

By jointly analyzing the quantitative and qualitative evidence in Figure 4a–d, it is evident that the remaining performance bottleneck under increasing occlusion is dominated by occlusion-induced fragmentation of visible evidence and the resulting spatial ambiguity, rather than a lack of category discriminability. Importantly, the proposed model maintains coherent spatial responses and usable detection outputs across occlusion regimes, while residual degradations mainly manifest as missed detections, localized bounding-box drift, and increased confidence sensitivity in occlusion-sensitive categories. The following comparative and ablation experiments further quantify how each proposed component contributes to mitigating these occlusion-driven failure modes.

### 5.2. Comparative Experiments

#### 5.2.1. Comparative Experiment 1: Comprehensive Performance Evaluation of CoFUSE-EPWNet and Mainstream Detectors Under Complex Occluded EPW Sorting Scenarios

This study evaluates seven representative object detectors to systematically assess the overall efficacy of CoFUSE-EPWNet in occlusion-intensive sorting scenarios of EPW. The baselines encompass both the two-stage detection paradigm (Faster R-CNN) and conventional single-stage lightweight architectures (SSD and the YOLOv5n–YOLOv12n series) [41,42,43,44,45]. All methods are evaluated under the same experimental settings, including dataset split, input resolution, training epochs, and evaluation metrics (See Table 6).

To make the comparison both interpretable and reproducible, this study uses a setting that combines single-modality reference models with modality-consistent multimodal comparisons. Faster R-CNN and SSD are used as RGB-only reference models with standard implementations and default configurations. These two models offer a reference point for understanding the normal operating range and failure modes of traditional detectors in EPW environments with high density stacking, multiple inner and outer packs and high occlusion. To make a fair multimodal comparison between YOLO series and the proposed method, synchronized RGB–depth inputs are used. By keeping Faster R-CNN and SSD as RGB-only references, the extra uncertainty from various RGB–depth fusion designs (alignment strategy, fusion level and depth-branch capacity) which can significantly impact both accuracy and reproducibility is avoided.

The results demonstrate that the traditional RGB-only detectors are limited in complex occlusion situations. Faster R-CNN and SSD achieve 50.1% and 53.9% ${mAP}_{50:95}$ with a strict IoU evaluation, which means that the models have poor localization consistency under strict evaluation. In comparison, CoFUSE-EPWNet achieves 69.3% ${mAP}_{50:95}$, which is 19.2% higher than Faster R-CNN and 15.4% higher than SSD. This improvement shows that the proposed method is more stable to localize EPW objects in the presence of occlusion and background clutter.

Due to the high accuracy of both the RGB–depth sensor and the trained network. The dual-modality YOLO baselines yield relatively high ${mAP}_{50:95}$ scores because both the RGB–depth sensor and the trained network are highly accurate, with values ranging from 85.4% to 86.4%. However, their recall and ${mAP}_{50:95}$ remain in a narrower range, with recall between 80.2% and 81.7% and ${mAP}_{50:95}$ between 62.7% and 65.9%. This indicates that simply adding depth information is not sufficient to solve occlusion-related problems, especially when visible evidence is fragmented and object boundaries are unclear. Taking YOLOv11n as the strongest YOLO baseline, it obtains 86.4% ${mAP}_{50}$, 65.9% ${mAP}_{50:95}$, 84.1% precision, and 81.7% recall. CoFUSE-EPWNet improves these values to 89.1%, 69.3%, 86.1%, and 85.1%, corresponding to absolute gains of 2.7, 3.4, 2.0, and 3.4 percentage points, respectively. These results show that, with the same synchronized RGB–depth input, the proposed method improves high-IoU localization and reduces missed detections while keeping precision at a competitive level.

CoFUSE-EPWNet requires 33 GFLOPs, which is about 1.74 times that of YOLOv11n with 19 GFLOPs. It also has 5.2 million parameters, about 2.08 times that of YOLOv11n with 2.5 million parameters. Although the computational cost is higher, the model gains 3.4 percentage points in both ${mAP}_{50:95}$ and recall. This suggests that the improvement mainly comes from the mechanism-oriented design for occlusion handling, rather than from simple model enlargement. In summary, CoFUSE-EPWNet offers a balance of accuracy and efficiency in EPW sorting scenes where accuracy and box drift directly impact sorting performance.

It should be noted that the baseline methods in Table 7 have different input-modality settings. Faster R-CNN and SSD are included as conventional RGB detection references, while the YOLO-series models and CoFUSE-EPWNet are evaluated under RGB-D input settings. Therefore, the comparison with Faster R-CNN and SSD is mainly used to show the performance difference between conventional RGB detectors and modern lightweight detectors in the proposed dataset, rather than to claim a strictly modality-equivalent comparison.

To further provide a fairer evaluation of the role of RGB-D input and fusion strategy, an input-modality comparison was conducted under a consistent detection framework. As shown in Table 7, the RGB-only setting achieves 83.8% AP50 and 63.5% AP50:95, while the depth-only setting obtains 78.2% AP50 and 57.6% AP50:95. This indicates that RGB appearance information provides stronger semantic cues than depth information alone, while depth maps still provide useful structural information for stacked packaging waste.

When RGB and depth images are jointly used, both early fusion and late fusion improve the detection performance compared with unimodal inputs. Early RGB-D fusion achieves 85.9% AP50 and 64.8% AP50:95, while late RGB-D fusion achieves 86.4% AP50 and 65.9% AP50:95. However, these simple fusion strategies still have limited ability to handle modality inconsistency caused by occlusion, deformation, reflection, and incomplete depth responses. In contrast, CoFUSE-EPWNet achieves 89.1% AP50 and 69.3% AP50:95, outperforming late fusion by 2.7 and 3.4 percentage points, respectively. These results indicate that the proposed cross-modal consistency fusion mechanism is more effective than direct or simple RGB-D fusion.

#### 5.2.2. Comparative Experiment 2: Inference Efficiency Comparison for Edge-Side Sorting

In addition to detection accuracy, inference efficiency is critical for real-time express packaging waste sorting [46]. Therefore, the per-frame inference time and FPS of representative detectors were further compared under the same hardware platform, input resolution, and batch size. The inference time was measured with batch size 1, excluding image loading time, so as to reflect the computational latency of model inference and post-processing.

As shown in Table 8, Faster R-CNN and SSD require 31.4 ms and 18.6 ms per frame, respectively, corresponding to 31.8 FPS and 53.8 FPS. Among the YOLO-series detectors, YOLOv11n achieves 8.1 ms per frame and 123.5 FPS. CoFUSE-EPWNet requires 13.8 ms per frame and achieves 72.5 FPS. Although the proposed model introduces additional cross-modal feature interaction and therefore has a slightly higher inference latency than lightweight YOLO-series models, it still satisfies the real-time inference requirement under the current prototype setting while achieving higher detection accuracy.

Furthermore, the lightweight CoFUSE-EPWNet achieves 7.6 ms per frame and 131.6 FPS after model compression. This indicates that the proposed framework can be further optimized for edge-side deployment when inference speed is prioritized. It should be noted that the inference time reported here only represents model-level computational latency. The complete system delay, including RGB-D image acquisition, preprocessing, PLC communication, actuator response, and mechanical grasping, is further analyzed in the system-level latency experiment.

#### 5.2.3. Comparative Experiment 3: Per-Category Detection Performance Analysis

To further analyze the detection behavior of CoFUSE-EPWNet across different types of express packaging waste, per-category AP50 and AP50:95 results were calculated on the test set. As shown in Table 9, CoFUSE-EPWNet achieves stable detection performance for most outer packaging categories. For example, PaperBox, FileSealing, WovenBag, and FoamBox obtain AP50 values of 95.8%, 93.4%, 94.1%, and 90.2%, respectively. These categories generally have more regular shapes, clearer object boundaries, and more stable depth structures, which makes them easier to localize under cluttered scenes.

In contrast, several inner cushioning materials show relatively lower AP50:95 values. For example, EPEFoam achieves 66.7% AP50 and 43.9% AP50:95, which is lower than most other categories. This is mainly because EPEFoam, AirBag, and BubbleFilm are often deformable, folded, semi-transparent, or reflective. Their boundaries are less stable in RGB images, and their depth responses may become incomplete or noisy when they are compressed, overlapped, or partially occluded. These characteristics make accurate localization more difficult, especially under stricter IoU thresholds.

Compared with the baseline YOLOv11n model, CoFUSE-EPWNet improves AP50 and AP50:95 across all categories. The improvement is particularly evident for soft cushioning materials. For EPEFoam, AP50 increases from 60.0% to 66.7%, and AP50:95 increases from 36.1% to 43.9%. For BubbleFilm, AP50:95 increases from 62.5% to 67.6%. These results indicate that the proposed cross-modal consistency fusion strategy can effectively exploit depth structure and RGB appearance information to improve detection robustness for deformable and occluded packaging waste.

#### 5.2.4. Comparative Experiment 4: Qualitative Visualization Comparison of CoFUSE-EPWNet and Mainstream Detectors in the EPW Sorting Scenarios with Different Occlusion Levels

**Figure 5:**
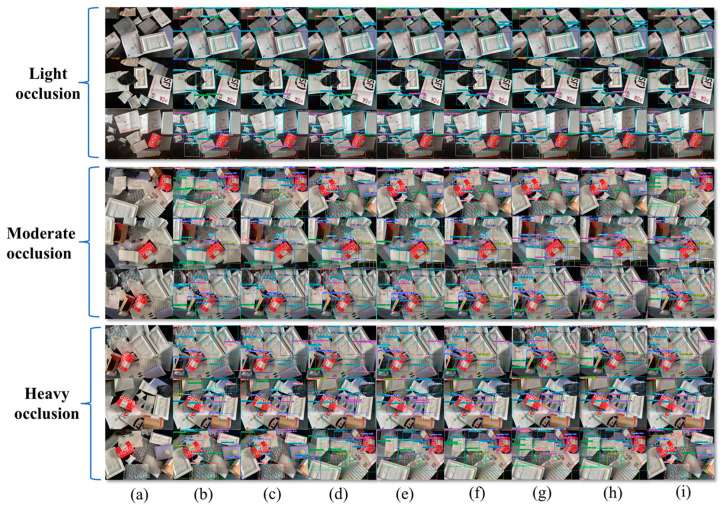
Object detection performance comparison. Visualization results from left to right: (**a**) original images; (**b**) Faster-RCNN; (**c**) SSD; (**d**) YOLOv5n; (**e**) YOLOv8n; (**f**) YOLOv10n; (**g**) YOLO11n; (**h**) YOLO12n; (**i**) ours.

Figure 5 shows a qualitative comparison of the various detection techniques in the EPW scenes with a high ratio of inner to outer packaging. Three failure modes that are relevant for engineering applications are investigated: occlusion that can cause failure in detection, drifting of BBoxes or poor fitting of BBoxes in crowded environments, and redundant BBoxes or adhesion of other objects on BBoxes, which can lead to false positive detections.

For the light-occlusion group, most of the mainstream models can identify the main targets, and the distinction is primarily on the stability of the boundaries. Faster R-CNN (Figure 5b) and SSD (Figure 5c) either have conservative boxes or local boundary drift. The predicted boxes can be affected by the presence of local high-contrast regions and thus may not be as correctly aligned when object edges are close to background textures, like printed text, folds or tape marks. Although the YOLO models (Figure 5d–h) alleviate this issue to a certain degree, duplicate boxes and box competition may still occur in regions with stacked boundaries and high content. This increases their sensitivity to NMS and confidences. The proposed method of Figure 5i, on the other hand, results in more stable object boundaries and fewer redundant detections. This agrees with the purpose of SS, which is to eliminate multi-scale spatial responses prior to fusion, whilst also eliminating interference from texture-driven responses, for cleaner predictions in real time sorting.

The moderate-occlusion group better reflects the core difficulty of EPW sorting. In these scenes, objects overlap heavily and their visible regions are fragmented, so the detector must recognize partially visible targets while avoiding adhesive boxes and broken predictions. As shown in Figure 5, Faster R-CNN and SSD (Figure 5b,c) are more likely to miss targets or produce discontinuous localization in densely stacked areas than the proposed method in Figure 5i. Some lightweight YOLO variants (Figure 5d–h) still achieve reasonable detection results, but they may generate one large box covering several neighboring objects or multiple competing boxes around the same object. These problems reduce the usefulness of the detection results for sorting. By comparison, Figure 5i shows more continuous detection of partially occluded objects, better boundary adherence in overlapping regions, and fewer adhesive or duplicate boxes. The improvement is closely related to the cooperation between IMR and SS. IMR suppresses unstable feature responses in each modality when occlusion or contamination weakens the input cues. SS further selects spatially consistent responses between RGB and depth features before fusion, which helps reduce confusion in densely stacked regions.

The heavy-occlusion group is for evaluating the model’s ability to cope with only a small amount of information about an object being visible. In these cases, targets might only be partially displayed or their edges might only be visible, resulting in a high number of missed detections and false positives in traditional algorithms. Faster R-CNN (Figure 4b) and SSD (Figure 5c) compared to Figure 5i have a tendency towards missing targets. The variants of YOLO (Figure 5d–h) are also able to detect some objects but are more sensitive to box drift and incompleteness of the boxes in dense clutter and redundant prediction. This indicates that these models might be too heavily dependent on the local sparse parts and thus experience a larger variation in localizations. By contrast, the proposed method in Figure 5i demonstrates that the proposed method is more stable under massive occlusion and is able to achieve more continuous detections with fewer false detections in textured and cluttered areas. This is part of PAH, which bundles patch-level evidence at the prediction stage to minimize the impact of “fractured visibility”. In combination with IMR and SS, it also offers more consistent and reliable features for the detection head, mitigating the missed detections and false positives under extreme occlusion.

This qualitative result in Figure 5 is in line with the quantitative comparison. This method achieves output purer without too many redundant boxes when occlusion occurs. It will decrease adhesion and boundary drift under moderate occlusion, rendering the results more suitable for sorting. It is more robust with fragmented visible evidence when under heavy occlusion. Combined, the observations suggest that the improvement is not just scaling up the model size, but rather a result of the combination of the design of IMR, SS and PAH.

To quantitatively evaluate the robustness of CoFUSE-EPWNet under different occlusion conditions, the test set was further divided into light-, moderate-, and heavy-occlusion groups according to the occlusion-level annotations defined in the dataset construction section. The baseline model and the proposed CoFUSE-EPWNet were evaluated separately on each occlusion group. AP50, AP50:95, precision, and recall were used as evaluation metrics.

As shown in Table 10, the detection performance of both models decreases as the occlusion level increases. This trend indicates that stacked objects, incomplete visible regions, and ambiguous boundaries increase the difficulty of express packaging waste detection. Under light occlusion, CoFUSE-EPWNet achieves 94.2% AP50 and 75.8% AP50:95. Under moderate occlusion, the model achieves 89.9% AP50 and 69.2% AP50:95. Under heavy occlusion, although the visible regions of packaging waste are severely reduced, CoFUSE-EPWNet still achieves 83.5% AP50 and 60.1% AP50:95.

Compared with the baseline model, CoFUSE-EPWNet consistently improves detection performance under all occlusion levels. The improvement is particularly evident under heavy occlusion, where AP50 increases from 78.9% to 83.5%, AP50:95 increases from 55.3% to 60.1%, and recall increases from 73.4% to 78.6%. This suggests that the proposed cross-modal consistency fusion mechanism can better exploit complementary RGB appearance cues and depth structural cues when target boundaries are incomplete or partially occluded.

#### 5.2.5. Comparative Experiment 5: Comparison with Representative RGB-D Fusion Baselines

To provide a more comprehensive comparison with multimodal detection methods, additional RGB-D fusion baselines were evaluated on the MEPWaste test set. In addition to RGB-only and depth-only detection, four multimodal settings were considered: early RGB-D fusion, late RGB-D fusion, cross-attention-based RGB-D fusion, and transformer-based RGB-D fusion. These baselines were evaluated under the same dataset partition, input resolution, and evaluation protocol.

As shown in Table 11, the RGB-only baseline achieves 83.8% AP50 and 63.5% AP50:95, while the depth-only baseline obtains 78.2% AP50 and 57.6% AP50:95. This indicates that RGB appearance features provide stronger semantic cues than depth information alone. However, depth information still provides useful structural cues for stacked and occluded packaging waste.

Compared with unimodal inputs, RGB-D fusion improves detection performance. Early fusion achieves 85.9% AP50 and 64.8% AP50:95, while late fusion achieves 86.4% AP50 and 65.9% AP50:95. Cross-attention-based and transformer-based RGB-D fusion further improve the AP50:95 to 66.5% and 66.8%, respectively. Nevertheless, these methods still show limited robustness under severe occlusion and boundary ambiguity because they mainly enhance feature interaction without explicitly addressing modality inconsistency and unreliable depth responses in cluttered packaging waste scenes.

In contrast, CoFUSE-EPWNet achieves 89.1% AP50 and 69.3% AP50:95, outperforming the transformer-based RGB-D fusion baseline by 1.6 percentage points in AP50 and 2.5 percentage points in AP50:95. This improvement demonstrates that the proposed cross-modal consistency fusion strategy is more effective for express packaging waste detection under stacking, occlusion, deformation, and complex background interference.

### 5.3. Ablation Study

Experiments including ablation were conducted to investigate the performance of CoFUSE-EPWNet in EPW sorting scenarios with mixed inner and outer packaging, severe occlusion and dense stacking. Performance is evaluated using P, R, ${mAP}_{50}$, and ${mAP}_{50:95}$ (Table 12). The challenges for EPW sorting are not just standard detection challenges, but also engineering challenges: occlusion fragments evidence into disjoint parts, stacking introduces spatial ambiguity and boundary adhesion, and surface artifacts (tape glare, printed text and folds) can cause texture-driven false activations. These failure modes directly carry over to downstream sorting resulting in missed picks, mis-grasps and unstable triggering. In this ablation study, the authors therefore investigate how IMR, SS and PAH can overcome these constraints to the EPW in a mechanism consistent way, and if their combination can create a closed loop in line with sortability.

(1) Modality configuration analysis. First, let us take a look at depth and its role in EPW sorting. For RGB-only input, compared to depth-only input, there is a significant drop ${mAP}_{50:95}$ by 16.1% and a significant drop in P and R. This validates that depth cues are not enough for EPW category discrimination, which is still largely based on appearance and texture cues. Combination of RGB and depth always outperforms the RGB only input by +2.4% for P, +1.8% for R, +2.6% for ${mAP}_{50}$ and +2.4% for ${mAP}_{50:95}$. The achieved improvements suggest that depth is not used as the sole discriminator in occluded regions of dense stacks, but rather that the geometric separability and boundary constraints are the primary advantages. Hence, the RGB–depth setting is used as the basic setting for module level ablations.

(2) Single-module ablation. Adding IMR on top of the RGB–depth baseline results in a reliability-driven improvement curve: P goes up 1.2%, R goes up 0.7%, ${mAP}_{50}$ goes up 0.8%, and ${mAP}_{50:95}$ goes up 0.9%. This is consistent with the EPW-specific noise sources, tape specularity, tape contamination and high-contrast printing, which can cause spurious channel responses and false-positives. IMR refines intra-modal channels, which helps to reduce unreliable activations and increase the likelihood of correct predictions in a cluttered sorting environment.

Adding SS gives a greater benefit, when localization requirements are strict. Compared with RGB–depth baseline, SS improves ${mAP}_{50:95}$ by 1.5% and ${mAP}_{50}$ by 1.1%, and P and R improve in a balanced manner by 1.3% and 1.1%. This helps to achieve the intended goal of SS—multi-scale spatial saliency selection and spatial alignment before fusion—to minimize the cross-modal spatial inconsistency and spatial localization drift in dense packaging positions where there is a high overlap of package boundaries and adhesion. This means better steady contact with the boundary and less adhesive boxes which affect grasp planning and object separation from an engineering point of view.

PAH has a very particular gain shape that addresses mainly the missed detections resulting from occlusion. Compared with the baseline, PAH increases R by 2.8%, ${mAP}_{50}$ by 1.9%, and ${mAP}_{50:95}$ by 0.5%. This means that PAH can reduce visibility fragmentation, that is, it can translate sparse cues (part of the edge, corner and texture) from a patch to more stable hypotheses (object) at the prediction stage. The smaller gap in ${mAP}_{50:95}$ indicates that despite the consistently strong high-IoU localization, complementary mechanisms that boost feature reliability and spatial coherence still help to improve the results.

(3) Two-module combinations. It is also found that there are obvious complementary effects when the various modules are combined. A result of 22.3% ${mAP}_{50:95}$ is achieved on IMR + SS, the highest among the two-module settings, a 2.5 percentage point increase from baseline. Meanwhile, precision and recall rise by 2.5 percentage points and 2.2 percentage points, respectively. This means that both feature reliability and boundary stability are improved, which is crucial for sorting applications as duplicate boxes, competing predictions and threshold-sensitive detections can impact downstream execution. On the other hand, SS + PAH primarily enhances the ability to discover objects when they are occluded. It improves ${mAP}_{50}$ by 2.3 percentage points and recall by 1.5 percentage points with respect to the baseline and increases ${mAP}_{50:95}$ by 1.3 percentage points. This indicates a better coverage and continuity, and a relatively lower localization in tight compact areas, which are densely stacked and close to the boundaries. IMR + PAH offers a better-balanced profile, a 1.8% higher ${mAP}_{50:95}$ and a 2.1% higher R, indicating that IMR helps to stabilize channel responses feeding patch-aware aggregation and is less volatile on prediction when there is little or disjointed evidence.

(4) Full model integration. CoFUSE-EPWNet is the optimum overall performance, having integrated IMR, SS and PAH. Compared to the RGB–depth base model, the entire model boosts ${mAP}_{50}$ by 2.7% and ${mAP}_{50:95}$ by 3.4%, while also improving P by 2.7% and R by 3.4%. The concurrent gains in precision and strict localization indicate fewer false triggers and more stable boundaries rather than over-detection. Overall, the ablation results support a mechanism-grounded closed loop for EPW sorting: IMR enhances channel reliability under contamination and specularity, SS enforces cross-modal spatial coherence prior to fusion, and PAH mitigates visibility fragmentation at the prediction stage—together yielding more sortable detections in dense, occluded EPW scenes.

### 5.4. Lightweight Ablation for Edge Deployment

Although CoFUSE-EPWNet improves the detection accuracy of express packaging waste under cluttered and occluded RGB-D scenes, its deployment in edge-side sorting equipment also requires a favorable balance between detection accuracy and computational efficiency. Therefore, this study further conducted a lightweight ablation analysis to evaluate the deployment potential of the proposed model. Three commonly used lightweight strategies were considered, including structured pruning, INT8 quantization, and knowledge distillation.

Structured pruning was used to remove redundant channels and low-contribution feature responses from the network, thereby reducing the number of parameters and computational cost. INT8 quantization compressed the floating-point weights and activations into low-precision integer representations, which reduced the model size and accelerated inference. Knowledge distillation used the original CoFUSE-EPWNet as the teacher model and a compact student network as the deployment-oriented model, so as to reduce accuracy degradation during model compression.

The lightweight ablation results are shown in Table 13. The original CoFUSE-EPWNet contains 5.2M parameters and 33.0 GFLOPs, with a model size of 10.6 MB and an inference time of 13.8 ms per frame. After structured pruning, the number of parameters decreases from 5.2M to 3.8M, and the inference time decreases from 13.8 ms to 10.5 ms. The corresponding AP50:95 decreases from 69.3% to 68.1%, with a decay of 1.2 percentage points. INT8 quantization reduces the model size from 10.6 MB to 3.1 MB and decreases the inference time to 8.7 ms, while the AP50:95 decreases by only 0.9 percentage points. Knowledge distillation further reduces the model complexity and maintains competitive detection performance, achieving 67.8% AP50:95 with 3.1M parameters.

After combining lightweight optimization strategies, the lightweight CoFUSE-EPWNet achieves 3.1M parameters, 18.9 GFLOPs, and a model size of 2.4 MB. The inference time decreases to 7.6 ms per frame, corresponding to 131.6 FPS. Compared with the original CoFUSE-EPWNet, the final lightweight version reduces model size by 77.4% and improves inference speed by 44.9%, while the AP50:95 decreases by only 2.2 percentage points. These results indicate that the proposed model can be compressed for edge-side deployment while maintaining acceptable detection accuracy.

### 5.5. Intelligent Detection and Automated Sorting System for Express Packaging Waste

**Figure 6:**
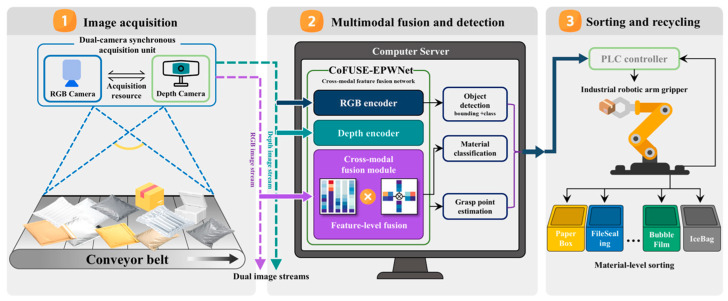
Intelligent system for the detection and automatic sorting for express packaging waste.

Figure 6 shows the prototype of an intelligent detection and automated sorting system for EPW under dense stacking and occlusion. The system forms a closed-loop workflow from multimodal sensing to physical sorting. It mainly includes synchronized RGB–depth acquisition, CoFUSE-EPWNet-based inference, and material-oriented sorting execution. An RGB camera and a depth camera are installed above the conveyor belt and triggered at the same time to collect paired RGB and depth images. This synchronized acquisition helps reduce motion-related mismatch during continuous conveying and provides aligned multimodal inputs for stacked and occluded EPW scenes.

Once acquired, the matched RGB and depth images are then passed to CoFUSE-EPWNet for real-time detection. The RGB branch aims to pull out visual features from the packaging such as color, texture, surface patterns to aid in distinguishing between various EPW categories. The depth branch provides shape and spatial information, particularly helpful if the packages overlap or if the boundaries of the objects are not well defined in the RGB image. The model then gives the position and category of each of the targets. These outputs are fed to the control unit to determine the sorting route, such as paper packaging, plastic film/bag material or cushioning material.

(1) Deployment and model efficiency at the edge: In the real world, the detection model must be continuously deployed in a sorting station with limited computing resources and strict real-time requirements. Hence, the deployment of the proposed system at the edge is an important factor. To reduce the computational cost, you can compress the model, reduce the cost of model inference, or convert the model in a lightweight way without affecting the overall detection pipeline. This optimization is helpful for conveyor belt sorting systems, as if it does not detect the object in time, the robotic arm may miss the correct grasping point. This will increase the system’s inference efficiency and facilitate the continuous EPW detection and sorting on resource-constrained edge devices.

(2) Camera placement and occlusion reduction: The placement of the camera directly impacts on the quality of the RGB–depth acquisition. Occlusion may also be increased and boundary estimation can be complicated by an inappropriate viewpoint when objects are stacked, folded, or partially covered by other packaging materials in EPW sorting. The RGB and depth cameras in the prototype system are placed above the conveyor belt to capture an overhead or top-down (or near top-down) view of the sorting area. This position minimizes mutual occlusion between objects and can reveal more complete contours of objects than low-angle views. In actual application, the camera height, viewing angle and sensing range need to be adjusted based on the width of the conveyor, the size of the object and the working distance, in order to guarantee the stable exposure of the sorting area.

(3) System latency and data transmission: In an automated sorting line, the time it takes for the image to be captured, model inferences to be made and the robot to execute should be kept to an acceptable time. Network instability during data transfer to a remote server for processing may impact real-time sorting performance if all image data are transferred to that server. Hence, edge computing architecture would be a better solution for EPW sorting applications. In this design, the RGB–depth data are processed locally at the detection unit, and only structured data indicative of object category, the location of the bounding-box of the object, the object confidence value, and sorting command is sent to the controller or management platform. This helps to decrease the bandwidth demand and increase the responsiveness of the system. This ensures stable operation of the sorting pipeline even as it is fed with constant input and heavy flow of objects.

### 5.6. System-Level Latency Analysis

(1) System-level latency analysis

Although model inference speed is an important indicator for edge-side deployment, the practical efficiency of an express packaging waste sorting system also depends on the complete operation pipeline. Therefore, this study further analyzed the system-level latency of the prototype sorting workflow. The measured stages include RGB-D data acquisition, preprocessing, model inference, post-processing, PLC communication, actuator response, and mechanical grasping.

As shown in Table 14, the RGB-D data acquisition stage requires 33.2 ms on average, including synchronized RGB and depth image capture. The preprocessing stage, including image alignment, resizing, and normalization, requires 7.8 ms. The lightweight CoFUSE-EPWNet requires 7.6 ms for model inference, and post-processing, including non-maximum suppression and category-to-command mapping, requires 4.5 ms. The PLC communication stage requires 9.2 ms, while the actuator response and mechanical grasping stages require 46.5 ms and 381.4 ms, respectively.

The total average cycle time of the prototype system is 490.2 ms. The results indicate that the visual perception module, including preprocessing, inference, and post-processing, accounts for only a small proportion of the total delay. In contrast, the mechanical grasping process is the dominant source of system latency. Therefore, the proposed visual detection framework satisfies the real-time perception requirement under the current prototype setting, while further improvement of system throughput should focus on mechanical execution, motion planning, and actuator control.

(2) Prototype sorting performance evaluation

To further evaluate the practical feasibility of the proposed framework beyond offline detection accuracy, a prototype-level sorting experiment was conducted under the controlled RGB-D sorting platform. The evaluation considered not only whether the object category was correctly detected, but also whether the detection result could be correctly converted into a sorting command and whether the mechanical execution was successfully completed. Sorting accuracy, detection-to-command accuracy, grasping success rate, throughput, and failure rate were used as system-level indicators.

As shown in Table 15, the prototype system achieved 84.6% sorting accuracy and 87.9% detection-to-command accuracy under the current experimental setting. The grasping success rate was 88.3%, indicating that most correctly detected objects could be successfully picked or transferred by the mechanical unit. The average system latency was 490.2 ms, and the corresponding throughput reached 108 objects per minute under the tested conveyor-belt configuration. These results suggest that the proposed RGB-D perception framework can support prototype-level real-time sorting of express packaging waste.

The failure rate was 11.7%. Failure cases were mainly associated with three situations. First, heavily occluded objects sometimes provided only small visible regions, resulting in missed detections or category confusion. Second, soft cushioning packaging materials such as BubbleFilm, AirBag, and EPEFoam often had ambiguous boundaries, deformation, or unstable depth responses, which caused localization drift. Third, film-like and air-filled packaging materials were more difficult to grasp due to slipping, deformation, or unstable contact during mechanical execution. These results indicate that future improvement should not only focus on detection accuracy, but also on grasping strategy, end-effector design, and long-term system stability.

## 6. Conclusions, Results, Limitations, and Future Studies

This paper aims at exploring the multimodal EPW detection in sorting scenarios involving both inner and outer packaging, dense packing and high occlusion. Both the detection robustness and applicability are assessed using experiments conducted with the MEPWaste dataset. The following are the main conclusions.

(1) The building of the MEPWaste dataset and its value. In this work we develop and re-release MEPWaste, a synchronized and spatially aligned RGB–depth paired dataset for EPW sorting with the use of a conveyor belt. Practical disturbances like random stacking, diverse packaging structures and various occlusion levels are included in the dataset. Hence, it is a good indicator to assess the detection robustness and the sorting feasibility in realistic EPW recycling scenarios.

(2) Design and performance of CoFUSE-EPWNet. In this work CoFUSE-EPWNet is developed for EPW detection by incorporating both RGB appearance information and depth based spatial information. This helps to achieve better boundary fits and continuity of boundaries in densely stacked scenes and minimizes the impact of depth noise, spatial mismatch and ambiguous fusion responses.

(3) The effectiveness of the channel–spatial–prediction mechanism. Comparative experiments and ablation studies reveal that the IMR–SS–PAH mechanism tackles three typical failure sources found in EPW detection: unreliable feature response, contamination; spatial inconsistency, under stacking; and fragmented visual evidence at prediction stage. These modules, when combined, make it possible to improve strict localization quality and recall, resulting in more stable detection results when facing heavy occlusion.

(4) System-level value for closed loop EPW sorting. This paper also introduces a closed-loop perception-to-sorting pipeline which integrates synchronized RGB–depth sensing, real-time multimodal inference and material-based routing. The pipeline performs the following functions: converting the detection results into sorting commands, and demonstrating the use of the proposed method in continuous conveyor-belt EPW sorting operations. From the perspective of green campus management, this closed-loop detection and sorting pipeline can further support campus-level recyclable-material recovery, waste-flow monitoring, and data-driven environmental management in parcel-intensive scenarios.

Future work will be conducted according to a phased and implementable plan. First, the MEPWaste dataset will be further expanded by collecting samples from more campus recycling scenarios, including courier stations, dormitory pickup areas, teaching-building recycling points, and temporary outdoor collection sites. More complex conditions, such as illumination variation, surface contamination, package deformation, severe stacking, and partial target truncation, will be included to improve dataset diversity and model generalization.

Second, the proposed prototype will be tested in long-term semi-real or real campus recycling environments. In addition to detection accuracy, future evaluation will include sorting accuracy, grasping success rate, throughput, failure modes, and maintenance stability. This stage will help verify whether the proposed RGB-D perception framework remains robust under continuous operation and more complex material-flow conditions.

Third, edge-side deployment will be further optimized. Lightweight strategies, including structured pruning, INT8 quantization, and knowledge distillation, will be integrated into embedded computing platforms such as industrial edge GPUs or Jetson-based devices. The goal of this stage is to reduce model size, inference latency, and energy consumption while maintaining acceptable detection accuracy for real-time sorting.

Finally, an active learning mechanism will be developed to support continuous model improvement. Misclassified samples, missed detections, localization-drift cases, and grasping-failure samples collected during prototype operation will be re-annotated and added to the training set. Through iterative model updating, the system is expected to gradually improve its robustness to unseen packaging types, complex occlusion, and dynamic sorting environments.

## Figures and Tables

**Table 1:** Dataset partition of MEPWaste.

Category	Type	Training Instances	Validation Instances	Test Instances	Total Instances	Proportion
PaperBox	Outer packaging	1215	174	347	1736	15.25%
FileSealing	Outer packaging	717	102	205	1024	8.99%
PlasticBag	Outer packaging	906	130	259	1295	11.38%
WovenBag	Outer packaging	568	81	163	812	7.13%
FoamBox	Outer packaging	1109	158	317	1584	13.91%
GasColumnBag	Inner cushioning	683	98	194	975	8.57%
AirBag	Inner cushioning	769	110	219	1098	9.65%
EPEFoam	Inner cushioning	519	74	149	742	6.52%
BubbleFilm	Inner cushioning	1169	167	334	1670	14.67%
IceBag	Inner cushioning	314	45	89	448	3.94%

**Table 2:** Distribution of samples and object instances under different occlusion levels.

Occlusion Level	Definition	RGB-D Pairs	Proportion	Object Instances	Instance Proportion
Level 1	Slight occlusion or relatively isolated objects with clear boundaries	1628	35.95%	3365	29.56%
Level 2	Moderate stacking or partial overlap with disturbed boundaries	1837	40.57%	4825	42.38%
Level 3	Severe stacking, strong boundary ambiguity, or large-area occlusion	1063	23.48%	3194	28.06%
Total	—	4528	100.00%	11,384	100.00%

**Table 3:** Occlusion-level distribution in the test set.

Occlusion Level	Test RGB-D Pairs	Test Instances	Proportion of Test Instances	Occlusion Level
Level 1	326	673	29.57%	Level 1
Level 2	367	965	42.40%	Level 2
Level 3	212	638	28.03%	Level 3
Total	905	2276	100.00%	Total

**Table 4:** Dataset partition of MEPWaste.

Subset	RGB-D Pairs	RGB Images	Depth Images	Proportion
Training set	3170	3170	3170	70.0%
Validation set	453	453	453	10.0%
Test set	905	905	905	20.0%
Total	4528	4528	4528	100.0%

**Table 5:** Experimental environment and parameters.

Hardware environment	CPU	15 vCPU AMD EPYC 7642 48-Core Processor
	RAM	32 GB
	Video memory	24 GB
Software environment	Operating System	Ubuntu 20.04.4 LTS
	GPU	NVIDIA GeForce RTX 3090
	Python	3.8.10
	Pytorch	Pytorch 2.0.1
	CUDA	12
	cuDNN	8.7
Parameters	Size of input images	640 × 640
	Learning rate	0.001
	Epochs	100
	Batch size	16
	Decay	0.0005

**Table 6:** Values of the mAP and recall rate of different models on the EPWaste dataset.

Method	${mAP}_{50}$(%)	${mAP}_{50:95}$(%)	Precision (%)	Recall (%)	Params (M)	GFLOPs	Size (MB)
Faster R-CNN	70.1	50.1	67.7	68.7	41.3	156.7	315.8
SSD	74.8	53.9	73.8	70.1	24.8	87.9	189.4
YOLOv5n	85.5	63.1	83.3	80.8	2.2	19.8	4.7
YOLOv8n	86.1	65.3	83.6	81.2	2.8	24.8	5.8
YOLOv10n	85.4	62.7	82.8	80.2	2.7	19.7	5.4
YOLOv11n	86.4	65.9	84.1	81.7	2.5	19	5.2
YOLOv12n	85.7	64.7	84.5	80.5	2.1	16.6	4.6
Ours (CoFUSE-EPWNet)	89.1	69.3	86.8	85.1	5.2	33	10.6

**Table 7:** Input-modality comparison under a consistent detection framework.

Setting	Input Modality	Fusion Strategy	Precision/%	Recall/%	AP50/%	AP50:95/%
RGB-only baseline	RGB	—	81.7	79.4	83.8	63.5
Depth-only baseline	Depth	—	76.5	74.1	78.2	57.6
Early-fusion baseline	RGB-D	Input-level concatenation	83.4	80.6	85.9	64.8
Late-fusion baseline	RGB-D	Feature-level fusion	84.1	81.7	86.4	65.9
CoFUSE-EPWNet	RGB-D	Cross-modal consistency fusion	86.8	85.1	89.1	69.3

**Table 8:** Values of the mAP and recall rate of different models on the EPWaste dataset.

Method	Input Modality	Inference Time/ms	FPS
Faster R-CNN	RGB	31.4	31.8
SSD	RGB	18.6	53.8
YOLOv5n	RGB-D	8.9	112.4
YOLOv8n	RGB-D	9.6	104.2
YOLOv10n	RGB-D	8.3	120.5
YOLOv11n	RGB-D	8.1	123.5
YOLOv12n	RGB-D	7.9	126.6
CoFUSE-EPWNet	RGB-D	13.8	72.5

**Table 9:** Values of the mAP and recall rate of different models on the EPWaste dataset.

Category	Type	Baseline AP50/%	CoFUSE AP50/%	AP50 Gain	Baseline AP50:95/%	CoFUSE AP50:95/%	AP50:95 Gain
PaperBox	Outer packaging	94.4	95.8	+1.4	76.1	77.5	+1.4
FileSealing	Outer packaging	91.4	93.4	+2.0	72.9	74.8	+1.9
PlasticBag	Outer packaging	88.8	91.0	+2.2	69.3	71.5	+2.2
WovenBag	Outer packaging	92.8	94.1	+1.3	73.4	75.0	+1.6
FoamBox	Outer packaging	88.7	90.2	+1.5	70.6	72.2	+1.6
GasColumnBag	Inner cushioning	86.2	89.8	+3.6	67.0	70.8	+3.8
AirBag	Inner cushioning	81.3	84.7	+3.4	58.6	62.8	+4.2
EPEFoam	Inner cushioning	60.0	66.7	+6.7	36.1	43.9	+7.8
BubbleFilm	Inner cushioning	84.3	88.4	+4.1	62.5	67.6	+5.1
IceBag	Inner cushioning	96.1	96.5	+0.4	72.5	76.9	+4.4
All classes	—	86.4	89.1	+2.7	65.9	69.3	+3.4

**Table 10:** Detection performance under different occlusion levels.

Occlusion Level	Method	Precision/%	Recall/%	AP50/%	AP50:95/%
Light occlusion	Baseline	88.6	86.9	91.8	72.4
Light occlusion	CoFUSE-EPWNet	90.5	89.7	94.2	75.8
Moderate occlusion	Baseline	83.9	81.5	86.7	65.6
Moderate occlusion	CoFUSE-EPWNet	86.7	85.0	89.9	69.2
Heavy occlusion	Baseline	76.8	73.4	78.9	55.3
Heavy occlusion	CoFUSE-EPWNet	81.2	78.6	83.5	60.1

**Table 11:** Detection performance under different occlusion levels.

Method	Input Modality	Backbone/Fusion Type	Precision/%	Recall/%	AP50/%
YOLOv11n-RGB	RGB	RGB-only detection	81.7	79.4	83.8
YOLOv11n-D	Depth	Depth-only detection	76.5	74.1	78.2
YOLOv11n-EF	RGB-D	Early fusion	83.4	80.6	85.9
YOLOv11n-LF	RGB-D	Late fusion	84.1	81.7	86.4
YOLO-RGBDtea	RGB-D	YOLOv7-based RGB-D fusion	84.8	82.4	87.1
MM-Net	RGB-D	Multimodal multi-scale fusion	85.3	83.1	87.6
CoFUSE-EPWNet	RGB-D	Cross-modal consistency fusion	86.8	85.1	89.1

**Table 12:** Results of ablation experiments involving the CoFUSE-EPWNet model on the MEPWaste dataset.

DAPG	CPDF	MS-SEAMHead	F1-Score	${mAP}_{50}$(%)	${mAP}_{50:95}$	Param (M)	GFLOPs
			69.3	73.2	55.1	2.6	6.7
√			70.4	74.0	55.4	4.1	7.4
	√		70.7	74.3	55.7	4.4	7.7
		√	70.2	73.7	55.1	4.3	7.9
√	√		71.4	75.0	56.3	6.6	9.6
√		√	71.2	74.8	56.1	6.1	9.3
	√	√	71.0	74.7	56.0	5.6	8.6
√	√	√	72.5	75.0	59.2	12.4	17.9

**Table 13:** Lightweight ablation results of CoFUSE-EPWNet.

Model Variant	Params/M	FLOPs/G	Model Size/MB	Inference Time/ms	FPS	AP50/%	AP50:95/%	AP50:95 Decay
Original CoFUSE-EPWNet	5.2	33.0	10.6	13.8	72.5	89.1	69.3	—
Structured pruning	3.8	24.6	7.9	10.5	95.2	88.2	68.1	−1.2
INT8 quantization	5.2	33.0	3.1	8.7	114.9	88.5	68.4	−0.9
Knowledge distillation	3.1	18.9	6.4	9.2	108.7	88.0	67.8	−1.5
Lightweight CoFUSE-EPWNet	3.1	18.9	2.4	7.6	131.6	87.6	67.1	−2.2

**Table 14:** System-level latency of the prototype sorting workflow.

Stage	Description	Mean Time/ms	Std./ms
RGB-D data acquisition	Synchronized RGB and depth image capture	33.2	2.5
Preprocessing	Image alignment, resizing, and normalization	7.8	1.1
Model inference	Lightweight CoFUSE-EPWNet forward inference	7.6	0.8
Post-processing	NMS and category-to-command mapping	4.5	0.6
PLC communication	Transmission of sorting command to actuator	9.2	1.4
Actuator response	Mechanical response and positioning	46.5	5.2
Mechanical grasping	Picking and placing operation	381.4	32.6
Total cycle time	Complete perception-control-grasping cycle	490.2	38.1

**Table 15:** Prototype sorting performance under the controlled RGB-D sorting setting.

Indicator	Result	Description
Sorting accuracy	84.6%	Proportion of objects correctly sorted into the target category
Detection-to-command accuracy	87.9%	Proportion of detection results correctly converted into sorting commands
Grasping success rate	88.3%	Proportion of objects successfully picked or transferred after command execution
Average system latency	490.2 ms	Average time of the complete perception-control-grasping cycle
Processing throughput	108 objects/min	Average number of objects processed per minute under the prototype setting
Failure rate	11.7%	Proportion of failed sorting or grasping attempts

## Data Availability

The data mentioned in this paper are available on request from the corresponding author.

## Abbreviations

**Abbreviation**	**Full name**
**EPW**	Express Packaging Waste
**RGB-D**	Red, Green, Blue and Depth
**MEPWaste**	Multimodal Express Packaging Waste dataset
**CoFUSE-EPWNet**	Cross-Modal Consistency Fusion Network for Express Packaging Waste Detection
**DAPG**	Depth-Aware Prior Guidance
**CPDF**	Cross-Modal Progressive Dynamic Fusion
**MS-SEAMHead**	Multi-Scale Spatial Enhancement and Attention Matching Head
**AP50**	Average Precision at IoU = 0.50
**AP50:95**	Average Precision averaged over IoU thresholds from 0.50 to 0.95
**FPS**	Frames Per Second
**FLOPs**	Floating-point Operations
**IoU**	Intersection over Union
**NMS**	Non-maximum Suppression
**PLC**	Programmable Logic Controller
